# Isavuconazole in the Treatment of *Aspergillus fumigatus* Fracture-Related Infection: Case Report and Literature Review

**DOI:** 10.3390/antibiotics11030344

**Published:** 2022-03-05

**Authors:** Beatrijs Mertens, Ruth Van Daele, Melissa Depypere, Katrien Lagrou, Yves Debaveye, Joost Wauters, Stefaan Nijs, Willem-Jan Metsemakers, Isabel Spriet

**Affiliations:** 1 Department of Pharmaceutical and Pharmacological Sciences, KU Leuven, B-3000 Leuven, Belgium; ruth.vandaele@uzleuven.be (R.V.D.); isabel.spriet@uzleuven.be (I.S.); 2 Pharmacy Department, University Hospitals Leuven, B-3000 Leuven, Belgium; 3 Clinical Department of Laboratory Medicine, University Hospitals Leuven, B-3000 Leuven, Belgium; melissa.depypere@uzleuven.be (M.D.); katrien.lagrou@uzleuven.be (K.L.); joost.wauters@uzleuven.be (J.W.); 4 Belgian National Reference Center for Mycosis, University Hospitals Leuven, B-3000 Leuven, Belgium; 5 Department of Microbiology, Immunology and Transplantation, KU Leuven, B-3000 Leuven, Belgium; 6 Department of Cellular and Molecular Medicine, KU Leuven, B-3000 Leuven, Belgium; yves.debaveye@uzleuven.be; 7 Intensive Care Unit, University Hospitals Leuven, B-3000 Leuven, Belgium; 8 Medical Intensive Care Unit, University Hospitals Leuven, B-3000 Leuven, Belgium; 9 Department of Development and Regeneration, KU Leuven, B-3000 Leuven, Belgium; stefaan.nijs@uzleuven.be (S.N.); willem-jan.metsemakers@uzleuven.be (W.-J.M.); 10 Department of Trauma Surgery, University Hospitals Leuven, B-3000 Leuven, Belgium

**Keywords:** fracture-related infection, osteomyelitis, *Aspergillus*, invasive aspergillosis, antifungal treatment, isavuconazole, bone penetration

## Abstract

*Aspergillus* fracture-related infection (FRI) is a rare, but severe complication in trauma surgery. The optimal antifungal treatment for *Aspergillus* osteomyelitis, including FRI, has not been established yet, as only cases have been documented and data on bone penetration of antifungal drugs are scarce. We describe a patient with *Aspergillus fumigatus* FRI of the tibia who was treated with isavuconazole after developing liver function disturbances during voriconazole therapy. Isavuconazole, the active moiety formed after hydrolysis of the prodrug isavuconazonium sulfate by plasma esterases, was administered in a maintenance dose of 200 mg q24 h, followed by 150 mg q24 h. The patient completed a six-month antifungal treatment course. Although fracture union was not achieved during six months of follow-up after therapy cessation, no confirmatory signs of FRI were observed. Additionally, two literature searches were conducted to review available data on antifungal treatment of *Aspergillus* osteomyelitis and bone penetration of antifungals. One hundred and eight cases of *Aspergillus* osteomyelitis, including six (5.6%) FRI cases, were identified. Voriconazole and (lipid formulations of) amphotericin B were the most commonly used antifungals. In three (2.8%) cases isavuconazole was prescribed as salvage therapy. Data on antifungal bone penetration were reported for itraconazole, voriconazole, amphotericin B, anidulafungin and 5-fluorocytosin. Isavuconazole might be a promising alternative for the treatment of *Aspergillus* osteomyelitis. However, standardized case documentation is needed to evaluate the efficacy of isavuconazole and other antifungals in the treatment of *Aspergillus* osteomyelitis, including FRI.

## 1. Introduction

Fracture-related infection (FRI) is a challenging and severe complication in musculoskeletal trauma surgery, especially in cases of open fractures [1,2]. Infection incidences up to 30% have been reported in cases of severe injury [2]. FRI is predominantly caused by direct inoculation of microorganisms during trauma or during the insertion of orthopedic devices [1]. *Staphylococcus aureus*, coagulase-negative staphylococci and Enterobacterales are the most commonly isolated microorganisms causing FRI [1]. However, FRI caused by fungi, such as the *Candida* and *Aspergillus* species, are rare [3,4,5,6,7]. Importantly, until today, the term ‘*Aspergillus* FRI’ has not been described in the literature as a separate entity [8]. However, the general term ‘*Aspergillus* osteomyelitis’ has been used to document different types of bone infection caused by *Aspergillus* species, such as vertebral, skull base and sternal osteomyelitis, and FRI [9,10,11]. *Aspergillus fumigatus* is the most frequently isolated species, followed by *A. flavus* [9,10,11,12]. Cases of *Aspergillus* osteomyelitis have been described both in immunocompromised and immunocompetent patients, depending on the infection site and the infection mechanism [9,10,11,13]. Three major mechanisms contribute to the pathogenesis of *Aspergillus* osteomyelitis: haematogenous dissemination from a primary infection site, contiguous spread from an adjacent infection site of invasive aspergillosis, and direct inoculation of conidia, secondary to surgery, epidural injection or trauma [9,10,11,13].

The optimal antifungal treatment for *Aspergillus* osteomyelitis has not been established yet [9,10,11,14]. Different antifungal drugs have been used, including triazoles, conventional and lipid formulations of amphotericin B (AmB) and echinocandins. As comparative clinical trials for *Aspergillus* osteomyelitis are lacking, treatment guidelines predominantly rely on published cases and case series [13]. Voriconazole is currently recommended as the primary antifungal treatment for *Aspergillus* osteomyelitis and should be combined with surgical therapy where feasible, according to the practice guidelines for the diagnosis and management of aspergillosis of the Infectious Diseases Society of America (IDSA) [13]. Although the optimal treatment duration remains unclear, *Aspergillus* osteomyelitis often requires long-term antifungal treatment [9,12,13,15]. Due to the risk for adverse drug reactions and drug interactions, long-term therapy might be challenging with voriconazole. Therefore, alternative antifungal treatment options might be needed [13]. 

We describe a case of *A. fumigatus* FRI following a bifocal open tibia and fibula fracture in which isavuconazole was used as salvage therapy. Additionally, a literature review of publications on the antifungal treatment of *Aspergillus* osteomyelitis and spondylodiscitis and manuscripts on the bone penetration of antifungal drugs was conducted. 

## 2. Case Report

**Figure 1:**
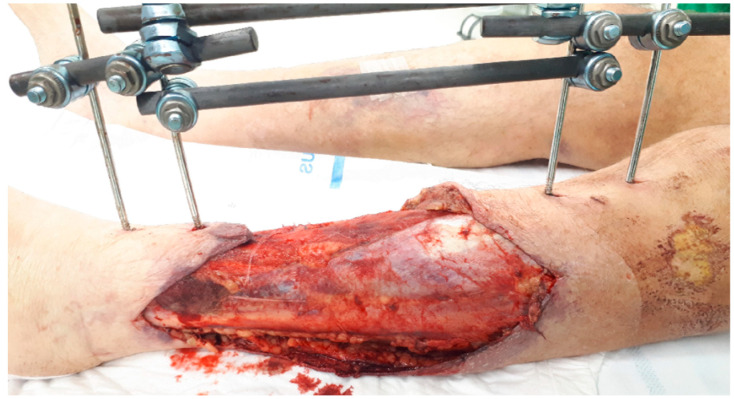
Clinical image of the bifocal Gustilo-Anderson type IIIB open tibia and fibula fracture, temporarily managed by external fixation (status on the fifth day after trauma).

This case report describes a 77-year-old male patient, weighing 88 kg, who was admitted to the emergency department after being involved in a car-on-bike collision. The patient’s medical background comprised of arterial hypertension, right-sided total hip arthroplasty and left-sided total knee arthroplasty. He sustained multiple injuries, including an intracerebral hemorrhage, fractures of the spine (D1, D5-D6), ribs, sternum, scapula and clavicle, and a bifocal Gustilo-Anderson type IIIB open tibia and fibula fracture on the left side (Figure 1). The open fracture was initially managed by external fixation and negative-pressure wound therapy (NPWT), followed by intramedullary nailing and reconstruction with a gracilis muscle flap and split-thickness skin graft approximately one month after admission. During the last NPWT dressing change in the operative room, five intra-operative deep-tissue specimens were taken. *A. fumigatus* was identified by culture from all specimens, confirming the diagnosis of an FRI [16,17]. Clinical confirmatory (e.g., purulent drainage and fistula) and suggestive criteria (e.g., redness and fever) were absent [16]. Laboratory analysis revealed a C-reactive protein (CRP) level of 65.1 mg/L and a white blood cell count (WBC) of 12.5 × 10^9^/L. No chest computed tomography (CT) scans were performed at the time of FRI diagnosis to evaluate haematogenous dissemination from a primary infection site (i.e., invasive pulmonary aspergillosis).

Intravenous voriconazole therapy was initiated in accordance with the IDSA practice guidelines for the diagnosis and management of aspergillosis [13]. No azole-resistance mechanisms were detected based on a validated matrix-assisted laser desorption/ionization time-of-flight (MALDI-TOF) mass spectrometry method, confirming susceptibility of the *A. fumigatus* isolates to voriconazole. The standard dosing regimen for invasive aspergillosis was prescribed (i.e., 6 mg/kg q12 h on day 1, followed by 4 mg/kg q12 h) [13]. Plasma trough concentrations between 2 mg/L and 5.5 mg/L were targeted in the absence of specific target values for bone infections [18]. Based on a previous report on voriconazole bone penetration, concentrations in this range were expected to correspond with bone exposure exceeding the European Committee on Antimicrobial Susceptibility Testing (EUCAST) breakpoint of 1 mg/L for *A. fumigatus* [19]. Plasma trough concentrations of 6.0 mg/L (day 4 after voriconazole initiation) and 6.5 mg/L (day 7) were measured, resulting in gradual dose reductions to 300 mg q12 h (day 7) and 150 mg q12 h (day 9). No cytochrome P450 (CYP450) pharmacokinetic interactions were detected and the CYPC219 ‘poor metabolizer’ phenotype was excluded. Despite therapeutic plasma trough concentrations of 4.1 mg/L and 4.3 mg/L on day 11 and 12, respectively, voriconazole was switched to isavuconazole after 13 treatment days due to a progressive increase in liver function parameters. A detailed graphical presentation of liver function parameters is provided in Appendix A. Shortly after voriconazole cessation, the patient was diagnosed with a cholangiocarcinoma, which was managed conservatively. Liver function parameters gradually decreased to a new baseline after voriconazole discontinuation, as shown in Appendix A.

Intravenous isavuconazole therapy was initiated according to standard dosing recommendations for invasive aspergillosis (i.e., loading dose of 200 mg q8 h for 48 h, followed by a maintenance dose of 200 mg q24 h) and switched to the oral capsule formulation on the 16th treatment day [13]. Plasma trough concentrations on day 4, 11, 20, 27, 29 and 34 of isavuconazole therapy were 2.3 mg/L, 3.9 mg/L, 4.3 mg/L, 3.7 mg/L, 4.7 mg/L and 6.6 mg/L, respectively. Although no upper limit for isavuconazole plasma exposure has been defined, the dose of isavuconazole was decreased to 100 mg q24 h (d36) to avoid plasma concentrations exceeding 5.0 mg/L [20,21,22]. Consecutively, plasma trough concentrations declined to 4.8 mg/L (day 40), 5.1 mg/L (day 43), 4.0 mg/L (day 47) and 2.7 mg/L (day 56). On day 57, isavuconazole dose was increased to an alternating regimen of 100 mg q24 h and 200 mg q24 h to target plasma concentrations in the upper part of the therapeutic range as the bone penetration of isavuconazole has not been defined thoroughly. Following dose augmentation, a plasma trough concentration of 3.2 mg/L was determined (day 63). During isavuconazole therapy, CRP gradually declined from 66.6 mg/L (day 4) to 13.5 mg/L (day 63). WBC evolved from 7.6 × 10^9^/L (day 4) to 5.3 × 10^9^/L (day 27), and then to 7.2 × 10^9^/L (day 63). Biomarkers, such as serum *Aspergillus* (galactomannan) antigen and 1,3-β-d-glucan, were not determined during antifungal treatment. 

After 97 hospitalization days, the patient’s medical condition was stable, and he was discharged to a nursing home. No clinical infections signs were observed at discharge. CRP corresponded to 13.5 mg/L and WBC to 7.7 × 10^9^/L. Radiographic imaging showed limited callus formation without osseous consolidation of the open tibia and fibula fracture. There were no signs of flap failure. No evidence of pulmonary dissemination was observed on a chest CT scan.

Ambulatory, follow-up visits were scheduled on days 92, 119 and 175 of isavuconazole treatment (i.e., days 29, 56 and 112 post-discharge). Isavuconazole trough concentrations were measured on days 92 and 119, corresponding to 1.7 mg/L and 2.8 mg/L, respectively (accidental interruption of isavuconazole therapy between treatment days 80 and 86). During follow-up visits, no local or systemic signs of FRI were observed clinically. Laboratory analyses showed CRP values of 9.9 mg/L and 9.8 mg/L and a WBC of 7.9 × 10^9^/L and 8.9 × 10^9^/L during the first two visits, respectively. Radiologically, any increase in callus formation was minimal without osseous consolidation. A positron-emission tomography-computed tomography (PET-CT) scan demonstrated areas of focal hypermetabolic activity located at the proximal and distal tibia fractures, which were suggestive for infection. Notwithstanding these findings, antifungal therapy was discontinued after 188 treatment days, considering a treatment duration of six months, the absence of confirmatory FRI signs and the decision for palliative management of the cholangiocarcinoma. At follow-visits during conservative FRI management (i.e., days 203 and 308 postdischarge) no clinical infection signs were observed, and standard X-rays revealed a progressive increase in callus formation with consolidation of the proximal tibia fracture, but no consolidation of the distal fracture. 

## 3. Methods Literature Review

Two literature searches were performed to review available data on (1) systemic antifungal treatment of *Aspergillus* osteomyelitis in adult patients and (2) bone penetration of antifungal agents with activity against *Aspergillus* species. 

### 3.1. Antifungal Treatment of Aspergillus Osteomyelitis

A PubMed search for English and Dutch manuscripts, published between January 2000 and January 2022, was conducted by combining the Medical Subject Headings (MeSH) and search terms “*Aspergillus*”, “Aspergillosis”, “Osteomyelitis”, “Bone and bones”, “Discitis”, “Spine”, “Epidural abscess” and “Fracture-related infection” with Boolean operators. All articles were screened for relevant information on systemic antifungal treatment of *Aspergillus* osteomyelitis and spondylodiscitis in adult patients. Additional studies were identified based on the reference lists of included papers. Articles were included if a conclusive diagnosis of *Aspergillus* osteomyelitis, including FRI, or spondylodiscitis was reported and information was given on the administered systemic antifungal treatment. The following exclusion criteria were applied: age < 18 years, animal data, cranial vault or skull base osteomyelitis. 

For all selected cases, information on demographics (age, sex), *Aspergillus* infection (type, location and mechanism of infection, isolated *Aspergillus* species), host factors, surgical treatment, antifungal treatment (treatment choice, posology and duration, rationale for antifungal therapy switch/discontinuation, therapeutic drug monitoring (TDM)), clinical outcome and duration of follow-up was collected. A detailed description of the applied terminology for the included variables is given in Appendix B. 

### 3.2. Antifungal Bone Penetration

A PubMed search of the English and Dutch literature, published before January 2022, was performed based on the advanced search term [(tissue*[ti] OR bone*[ti]) AND (“distribution”[tiab] OR “biodistribution”[tiab] OR “penetration”[tiab] OR concentration*[ti]) AND (“Antifungal Agents”[Mesh] OR antifungal*[tiab] OR “Itraconazole”[Mesh] OR “Voriconazole”[Mesh] OR posaconazol* OR isavuconazol* OR “Echinocandins”[Mesh] OR “Amphotericin B”[Mesh] OR “Flucytosine”[Mesh])]. All manuscripts were screened for eligibility and additional papers were selected based on the reference lists of included articles. Animal and human studies investigating bone (marrow) concentrations of antifungals with activity against *Aspergillus* species—itraconazole, voriconazole, posaconazole, isavuconazole, (lipid formulations of) AmB, caspofungin, anidulafungin, micafungin and 5-fluorocystosine (5-FC)—were included. 

## 4. Results Literature Review

### 4.1. Antifungal Treatment of Aspergillus Osteomyelitis

**Figure 2:**
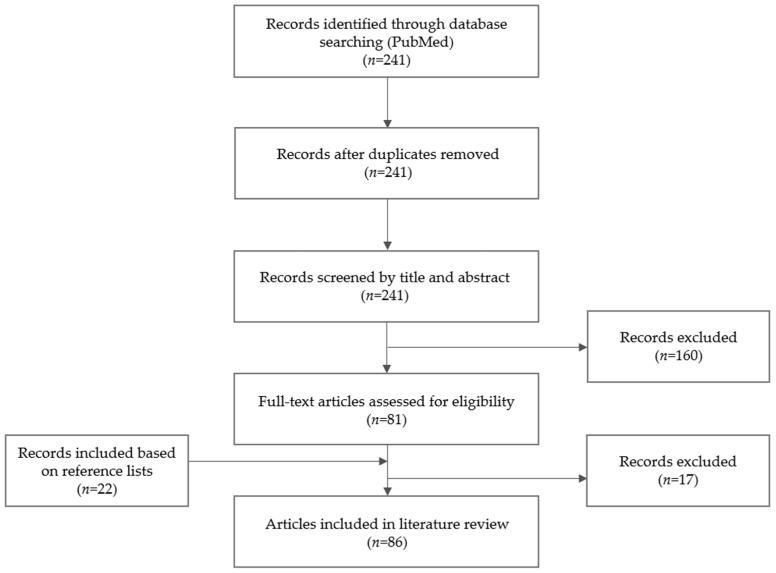
Selection process of articles on the antifungal treatment of *Aspergillus* osteomyelitis.

The PubMed search generated 241 records, of which 64 publications were included in the literature review. Additionally, 22 articles were identified based on the reference lists, resulting in a selection of 86 papers. A detailed overview of the article selection process is depicted in Figure 2. 

A total number of 108 *Aspergillus* osteomyelitis or spondylodiscitis cases wasidentified, including FRI (*n* = 6; 5.6%), rib/sternal osteomyelitis (*n =* 21; 19.4%), osteomyelitis of the lower extremities (*n* = 9; 8.3%), vertebral osteomyelitis (*n* = 39; 36.1%) and spondylodiscitis (*n* = 33; 30.6%). Demographic, clinical and therapeutic characteristics of *Aspergillus* FRI cases are summarized in Table 1. A comprehensive overview of all included cases is given in Appendix C. 

In all cases of *Aspergillus* FRI (*n* = 6), traumatic inoculation of conidia was documented as the origin of infection. It concerned patients without host factors, except for one patient with diabetes mellitus. In all cases, surgical and antifungal treatment was combined. Four patients received a single agent and two patients a sequential antifungal regimen. Voriconazole was administered in four cases, as primary or salvage therapy. Itraconazole and (liposomal) AmB were both used as part of a sequential treatment in two cases. The antifungal therapy duration varied from approximately 13 to 35 weeks. TDM was performed in two cases. In three cases, patients were reported to be infection free after a follow-up period ranging from four months to nine years. 

In 27 (25.0%) of all included cases (*n* = 108), contiguous/haematogenous spread was considered to be the origin of infection, compared to direct inoculation in 32 (29.6%) cases. The infection mechanism was not specified or unknown in 18 (16.7%) and 31 (28.7%) patients, respectively. In 55 (50.9%) patients, one or more host factors were identified. *A. fumigatus* was isolated in 63 (58.3%) infections, followed by *A. flavus* and *A. terreus* in 15 (13.9%) and 6 (5.6%) infections, respectively. In 80 (74.1%) patients, surgical treatment was combined with antifungal treatment. The triazoles voriconazole, itraconazole, posaconazole, isavuconazole and fluconazole were used in monotherapy or as part of sequential or combination therapy in 61 (56.4%), 37 (34.3%), 5 (4.6%), 3 (2.8%) and 2 (1.9%) patients, respectively. Fifty (46.3%) of the antifungal treatment regimens consisted of (lipid formulations of) AmB, of which nine were combination therapies with 5-FC. Thirteen (12.0%) patients were treated with echinocandins, which were part of a sequential treatment in 12 cases. In 18 (20.0%) patients treated with voriconazole, posaconazole or itraconazole (*n* = 90), TDM was performed. Detailed information on TDM in these cases is given in Table 2. The antifungal treatment duration varied from approximately one week to 22 months. Conclusive information on the infection outcome was described in 54 (50.0%) patients, of whom 25 died (14 related to invasive aspergillosis) and 23 were free of infection. The follow-up of infection-free patients ranged from 26 days to 9 years. 

### 4.2. Antifungal Bone Penetration

**Figure 3:**
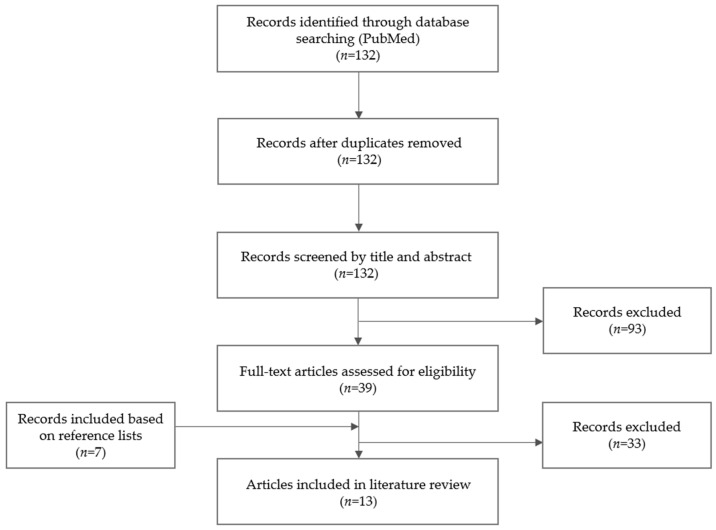
Selection process of articles on the bone penetration of antifungal agents.

One hundred and thirty-two records were identified based on the PubMed search, of which six were included in the literature review. Seven additional articles were selected based on the reference lists of included papers as depicted in the flow diagram of the article selection process (Figure 3). In total, 13 papers reported on the bone penetration of itraconazole, voriconazole, isavuconazole, AmB or anidulafungin. 

#### 4.2.1. Triazoles

Triazole antifungal agents inhibit the biosynthesis pathway of ergosterol, the sterol that regulates the permeability and fluidity of the fungal cell membrane, via inhibition of the enzyme 14-α-demethylase. The lack of ergosterol in the fungal cell membrane and the accumulation of toxic precursors causes fungal cell death [32].

##### Itraconazole

Itraconazole is a very lipophilic triazole (distribution coefficient (logD) of >5 at pH 7.4) with a volume of distribution (V_d_) of 11 L/kg that is strongly bound to plasma proteins (99.8%) [32,33,34]. The bone penetration of itraconazole is reported to be high, based on a bone/plasma concentration ratio of 4.7 measured after repeated oral administration of itraconazole in a single patient [32,33,34]. 

##### Voriconazole

Voriconazole is a relatively lipophilic molecule (logD of 1.8) with a plasma protein binding (PPB) of 58% and a V_d_ of approximately 4.6 L/kg. Therefore, it penetrates well into various tissues such as liver, lungs, spleen, kidneys, brain and myocardium [32,33,34]. Denes et al. determined voriconazole concentrations in medullar and cortical bone after an above-the-knee amputation in an 83-year-old woman with *A. fumigatus* arthritis and osteomyelitis who was treated intravenously with voriconazole (Appendix C) [19]. Concentrations of 20.3 µg/g and 1.9 µg/g were measured in medullar and cortical bone (day 6), respectively. Plasma trough concentrations of voriconazole, measured on the first two treatment days, were 2.41 mg/L and 4.09 mg/L, respectively. 

##### Posaconazole

Posaconazole is lipophilic triazole (logD of 2.15) with a high V_d_ (7–25 L/kg) and plasma protein binding (>98%) that distributes well into various organs and tissues, with the highest concentrations measured in the liver, followed by the kidneys, lungs and myocardium [32,33,34]. No data on the bone penetration of posaconazole have been reported in the literature. 

##### Isavuconazole

Only a paucity of data on the tissue distribution of isavuconazole (PPB: 98–99%,V_d_: 5.6 L/kg) in animals and humans is available, suggesting favorable penetration into brain tissue and soft tissue (muscle and fat) [32,34]. Bone penetration of isavuconazole has only been investigated in non-infected rats following single (5 mg/kg) and repeated (30 mg/kg q24 h for 21 days) oral administration of the prodrug isavuconazonium sulfate [35]. Following a single dose, a maximal isavuconazole bone concentration of 0.070 µg/g was measured after two hours. Bone concentrations determined at least four hours after single administration or concentrations measured on treatment day 1, 7, 14 and 21 following repeated dosing were below the limit of quantification or undetectable. 

#### 4.2.2. Echinocandins

Echinocandins cause depletion of the polysaccharide 1,3-β-D-glucan, an essential component for the integrity of the fungal cell wall, via inhibition of the enzyme 1,3-β-D-glucan synthase [32]. Caspofungin (logD: −3.88, V_d_: 0.15 L/kg, PPB: 97%), anidulafungin (logD: −3.32, V_d_: 0.8 L/kg, PPB: 84–99%) and micafungin (logD: −1.62, V_d_: 0.3 L/kg, PPB: >99%) have demonstrated a good penetration into the kidneys and alveolar cells, a variable distribution in liver and a poor penetration into the central nervous system, eyes, lung tissue and epithelial lining fluid [32,33,34]. Data on bone penetration of caspofungin, the only echinocandin that has been approved for the treatment of invasive aspergillosis, and micafungin are lacking. For anidulafungin, bone concentrations following subcutaneously administered single (10 mg/kg) or multiple doses (10 mg/kg q24 h for five days) have been measured in neonatal rats of postnatal age of four or eight days [36]. Single-dose administration of anidulafungin resulted in a bone/plasma concentration ratio of 0.95 and 1.2 in rats with a postnatal age of four and eight days, respectively. Following repeated-dose administration, a bone/plasma concentration ratio of 1.2 was measured on postnatal day 8. 

#### 4.2.3. Polyenes

AmB is a polyene that exerts antifungal activity by creating pores in the fungal cell membrane through interaction with ergosterol and by inducing oxidative cell damage [32]. AmB deoxycholate (d-AmB) and liposomal AmB (L-AmB), the most commonly used lipid formulation of AmB, are both characterized by a logD of −2.8 and a PPB of >95%, but a variable V_d_ (0.5–5.0 for D-AmB and 0.1–0.7 for L-AmB) [32,33,34]. The tissue concentrations of D-AmB and L-AmB are highest in liver and spleen, intermediate in lung and kidneys, and low in myocardium and brains [32]. Bone penetration of AmB has only been investigated for the deoxycholate formulation in non-human primates and was found to be limited [37]. D-AmB concentrations of 0.02–0.04 µg/g were measured in femoral bones 24 h after single intravenous administration (1 mg/kg) in two female rhesus macaques compared to serum concentrations of 0.73–1.73 mg/L. 

The penetration of different AmB formulations into bone marrow has been evaluated in rat and dog models. In the study by Groll et al., bone marrow concentrations of D-AmB (1 mg/kg q24 h for seven days) and AmB lipid formulations (L-AmB, AmB lipid complex (ABLC) or AmB colloidal dispersion (ABCD); 5 mg/kg q24 h for seven days) were measured in rats 30 min after the last administration [38,39]. The lowest tissue/plasma concentration ratio of 0.7 was obtained for L-AmB compared to 5.7 for D-AmB, 42.1 for ABLC and 54.7 for ABCD. In a dog model, Fielding et al. demonstrated higher bone marrow concentrations for ABCD compared to D-AmB. Bone marrow concentrations, measured 48 h after 14 consecutive daily doses, were 7.5 µg/g and 96 µg/g for ABCD in a dosage of 0.6 mg/kg q24 h and 5.0 mg/kg q24 h, respectively, and 2.7 µg/g for D-AmB in a dosage of 0.6 mg/kg q24 h [40].

Penetration of D-AmB and ABCD into the intervertebral disc space has been studied by Conaughty et al. in a non-infected adult male rabbit model [41]. Limited bioavailability in the nucleus pulposus was demonstrated after intravenous administration of D-AmB and ABCD (1 mg/kg q24 h and 5 mg/kg q24 h for two days, respectively). No detectable levels of D-AmB were found in the nucleus pulposus samples. For ABLC, a concentration of 14.53 µg/g was detected only in one rabbit. 

#### 4.2.4. 5-Fluorocytosine

5-FC is a prodrug that is converted into 5-fluorouracil, which is phosphorylated to 5-fluorouridine triphosphate. Following incorporation into fungal RNA, 5-fluorouridine triphosphate inhibits fungal protein synthesis [32]. 5-FC is a hydrophilic molecule (logD of –2.34) with a low PPB (5%) and a V_d_ of 0.6–2.2 L/kg that distributes well into various tissue fluids, such as cerebrospinal fluid, saliva, ascites and bronchial secretions [32,33]. In vivo biodistribution experiments in sarcoma-bearing rats have demonstrated a comparable uptake of [^18^F] 5-FC in blood and bone, two and four hours after a single injection of the radionuclide [42]. In the paper of Polak et al. on the pharmacokinetics of 5-FC in animals and humans a bone/plasma concentration ratio of 0.3 was documented [43].

## 5. Discussion

### 5.1. Antifungal Treatment of Aspergillus Osteomyelitis

We presented the first case report of a patient with *A. fumigatus* FRI who was treated with isavuconazole. This case report was complemented by a comprehensive review of the recent literature on antifungal treatment of *Aspergillus* osteomyelitis and spondylodiscitis, with a specific focus on *Aspergillus* FRI. Although this review yielded 86 manuscripts, describing 108 patients with *Aspergillus* osteomyelitis of whom six patients experienced a FRI, evidence remains limited to case reports and case series. Direct comparison of the published cases is challenging as the reported definitions (e.g., definition of osteomyelitis), treatment data (e.g., choice, posology and duration of antifungal treatment) and clinical outcome measures are highly heterogeneous or even inconclusive.

First, an extensive variability in the choice of antifungal agent has been reported [9,10,11,14]. Triazoles, deoxycholate and lipid formulations of AmB and echinocandins have been used as primary therapy or as part of a sequential antifungal treatment [3,4,5,6,12,15,19,23,24,25,26,27,28,29,30,31,44,45,46,47,48,49,50,51,52,53,54,55,56,57,58,59,60,61,62,63,64,65,66,67,68,69,70,71,72,73,74,75,76,77,78,79,80,81,82,83,84,85,86,87,88,89,90,91,92,93,94,95,96,97,98,99,100]. In the majority of cases described in our review, the antifungal regimen included voriconazole (56.4%), followed by AmB (46.3%) and itraconazole (34.3%). In the review by Gabrielli et al., voriconazole and AmB were used in 45% and 52% of 122 cases of *Aspergillus* osteomyelitis or spondylodiscitis, published between 2000 and 2013, respectively [10]. Direct comparison of their findings and ours is difficult due to differences in inclusion criteria—i.e., children and cases of skull base osteomyelitis were included in the review by Gabrielli et al. [10]. Nevertheless, voriconazole and AmB were the most commonly used antifungals for *Aspergillus* osteomyelitis in both reviews. AmB—alone or in combination with 5-FC—is no longer considered the drug of choice due to its limited bone penetration and the risk for renal and haematological toxicity [5,9,10,11,14,33,34,37,83]. Since its approval for the treatment of invasive aspergillosis in 2000, voriconazole has been increasingly used [10]. It is recommended as the primary antifungal drug for the treatment of *Aspergillus* osteomyelitis by the IDSA, based on the clinical experience with voriconazole in bone aspergillosis cases and the superior efficacy and safety profile compared to AmB in patients with invasive pulmonary aspergillosis [13,101]. The reported experience with posaconazole and echinocandins is limited and these antifungals have been used predominantly as part of a sequential treatment [26,27,28,31,47,49,54,62,70,81,82,84,91,93]. Although *Aspergillus* species are intrinsically resistant to fluconazole, in two case reports fluconazole was part of the antifungal regimen [49,60]. Isavuconazole has been described as a salvage therapy in only four cases, including in our patient [25,27,84].

In our case, voriconazole was used as primary therapy in accordance with the IDSA guidelines [13]. However, as liver function parameters progressively increased despite dose reductions guided by TDM, discontinuation was deemed necessary. Different alternative antifungal treatment options were evaluated by a multidisciplinary expert team. Considering the limitations of (long-term) L-AmB treatment (i.e., lack of an oral formulation, toxicity risk and limited bone penetration), isavuconazole was chosen as an alternative therapy based on its favorable safety profile, availability as an oral formulation and—albeit limited—clinical experience in the treatment of *Aspergillus* osteomyelitis [20,25,27,32,84]. Although the importance of combined surgical-pharmacological infection management has been highlighted in the IDSA guidelines, no surgical intervention was performed in our case due to the patient’s frailty [13]. The antifungal therapy was continued for approximately six months and no confirmatory signs of FRI were observed during follow-up. Six months after therapy discontinuation, no recurrence of infection was documented. However, as fracture union was still not observed, persistence of infection might be suspected. Our findings are in accordance with the report by Assaf et al. [25]. They described a patient with *A. fumigatus* sternal osteomyelitis who was treated with isavuconazole after experiencing adverse events during voriconazole and L-AmB treatment. A twelve-month antifungal treatment course resulted in full regression of the bone lesions and no recurrence was observed 12 months after therapy cessation. In contrast, a fatal outcome was documented in a transplant patient with *A. fumigatus* vertebral osteomyelitis who was treated with AmB, followed by a combination of isavuconazole and anidulafungin. This patient died due to progression of invasive aspergillosis [84]. Routray et al. described the need to switch isavuconazole therapy to L-AmB, followed by micafungin and voriconazole due to poor treatment adherence in a case of *A. fumigatus* sternal osteomyelitis [27]. As the patient’s follow-up was not clearly documented, interpretation of the clinical outcome should be done with caution. 

Second, a high degree of variability in the antifungal treatment duration has been documented. The therapy duration in our literature review varied from approximately one week to 22 months. This heterogeneity has been addressed previously in a review of 180 cases of *Aspergillus* osteomyelitis or spondylodiscitis, in which therapy duration ranged from 10 to 772 days [9]. A minimum antifungal treatment duration of eight weeks is recommended by the IDSA, but the evidence for this recommendation is limited [13]. The utility of serial serum 1,3-β-D-glucan monitoring to guide treatment duration has only been documented in a case report of *A. fumigatus* rib osteomyelitis treated with voriconazole and posaconazole [26]. Due to the limited evidence, the IDSA practice guidelines do not recommend routinely monitoring of serum 1,3-β-D-glucan [13]. The role of 1,3-β-D-glucan and other fungal biomarkers (e.g., galactomannan) to guide antifungal treatment of *Aspergillus* bone infections should be further scrutinized [13]. 

The heterogeneity of included case reports might be partially explained by the broad selection criteria that were applied in our literature review, especially with the inclusion of multiple types of osteomyelitis as well as spondylodiscitis. We comprehensively classified the selected case reports based on the infection type and documented the infection mechanism and host factors by using standardized definitions [102]. However, classification of the included cases was complex, as terminology used in *Aspergillus* osteomyelitis literature is largely ambiguous and inconclusive. *Aspergillus* osteomyelitis covers a diverse spectrum of infection types and standardized definitions are lacking, except for FRI and prosthetic joint infections (PJI) [16,103]. Confirmatory criteria for FRI, developed by an international expert panel, were used to diagnose FRI in our case report [16]. Furthermore, the consensus definitions of FRI and PJI were applied to standardize terminology in our overview of published cases. However, no cases of *Aspergillus* PJI were identified. 

Additionally, various clinical outcome measures were reported in the included case reports and multiple terms were used to describe clinical outcome (e.g., infection cure, complete/partial clinical cure, no recurrence of symptoms/infection). Furthermore, the duration of clinical follow-up was either highly variable or the follow-up information was inconclusive/lacking. Due to this methodological heterogeneity, treatment outcomes could not be documented conclusively in our literature review. Therefore, standardized documentation of the clinical outcome measures and follow-up duration, based on internationally developed criteria, should be encouraged. In our case report we provided a comprehensive overview of the results of clinical examinations, radiographic and PET-CT imaging and laboratory analyses at different time points. Given the importance of long-term surveillance for FRI, a follow-up of at least one year after antifungal therapy cessation is intended in our case, especially as fracture union was still not observed during the last follow-up visit [104]. For fungal FRI, long-term follow-up might be even more important due to the risk for slow onset of infection recurrence [8,105]. 

### 5.2. Antifungal Bone Penetration

In our literature review on the antifungal bone penetration, only 13 relevant publications (i.e., 10 original articles and 3 reviews) were included, reflecting the limited amount of data that is available. Important limitations of these studies should be addressed. First, five of the original reports were published before the year 1992 [37,40,42,43,106]. Second, in the majority of included studies, bone penetration was investigated in animal models, limiting the extrapolation of these data to humans [35,36,37,38,40,41,42]. In the report by Polak et al., it was not clear whether the reported bone/plasma concentration ratio of 5-FC was derived from animal or human studies [43]. Third, the generalizability of the results is challenging due to the limited sample sizes and methodological variability (e.g., heterogeneity in pharmacokinetic sampling, sample preparation, detection method) of the included studies. Importantly, a detailed methodological description was even absent in the reports by Heykants et al. and Polak et al. [43,106]. Fourth, in the majority of studies, bone concentrations were investigated in non-infected animals or humans, disregarding the effect of the infection state on bone penetration [35,36,37,38,40,41,42,106,107,108]. 

Furthermore, correct interpretation and direct comparison of reported bone concentrations is complex. First, concentrations are often determined in bone homogenates [33,108,109]. As (antifungal) drugs and pathogens do not uniformly distribute into the bone matrix, analyses of concentrations in bone homogenates might be of limited value. Cortical bone and cancellous bone are sometimes separated before homogenization due to differences in their composition [108]. Importantly, the nature of the tissue homogenate needs to be clearly specified to enable correct interpretation of documented bone concentrations [108,109]. Second, variability in bone concentration reporting has been documented [108]. Bone concentrations are predominantly expressed in relation to the total bone mass (i.e., µg/g of total bone mass). However, as other denominators are being used too (e.g., total dried bone mass, total bone volume, organic bone mass), caution is needed when interpreting and comparing bone concentrations. Third, bone concentrations are often expressed in relation to plasma concentrations. However, hysteresis—i.e., discordance in concentration–time profiles of drugs in plasma and bone—renders interpretation of bone/plasma concentration ratios challenging unless an equilibrium has been reached between both compartments [33,108,109]. For instance, in the case report by Denes et al. voriconazole plasma concentrations were measured on the first two treatment days, whereas bone concentrations were determined on the sixth day of therapy, leading to a non-reliable estimation of bone penetration [19]. Noteworthy, erroneous representation of bone/plasma concentration ratios, as in the review by Stover et al., might further increase the risk for misinterpretation [34]. Fourth, bone concentrations are often compared to minimal inhibitory concentrations of antifungals to isolated pathogens. The calculated quotient should, however, be interpreted cautiously as bone concentrations depend on various factors (e.g., timing of bone sampling) that are not captured by a single ratio [108]. 

As bone concentrations are difficult to determine in clinical practice, further research on the utility of plasma concentration monitoring for antifungals with adequately proven bone penetration might provide added value. In order to relate plasma concentrations and clinical outcomes, standardization of clinical outcome measures is needed, as well as adequate documentation of the measured plasma concentrations. Of note, in our literature review, TDM was reported in only 18 (20.0%) patients treated with itraconazole, posaconazole or voriconazole (*n* = 90) [3,4,15,18,19,23,24,25,26,27,28,29,30,31]. Information on blood sampling and measured plasma concentrations was, furthermore, inconclusive or lacking in a part of these TDM cases. 

### 5.3. Expert Opinion

Based on our review, itraconazole (*n* = 37), voriconazole (*n* = 61) and different formulations of AmB (*n* = 50) were used most frequently to tackle *Aspergillus* osteomyelitis. Although itraconazole is cheap and widely available, its use is hampered by its low bioavailability related to poor absorption, frequently leading to underexposure. AmB is often prescribed as initial treatment, but long-term use is impeded by the risk of nephrotoxicity and the lack of an oral formulation. Voriconazole, indicated by the IDSA guidelines as first-line treatment for invasive aspergillosis, is available as an oral formulation with high bioavailability, allowing long-term (ambulatory) treatment. Plasma concentrations of voriconazole, based on regular TDM, should be targeted in the higher range of the therapeutic window to ensure adequate bone exposure. Voriconazole treatment might be challenging due to its complex pharmacokinetics necessitating frequent monitoring of plasma exposure, involvement in drug-drug interactions and association with hepatotoxicity and neurotoxicity. Based on the limited experience, including our case report, isavuconazole might be a promising alternative for the treatment of *Aspergillus* osteomyelitis. In the case of documented azole resistance, lipid formulations of AmB or high-dose posaconazole, targeting a plasma trough concentration >3 mg/L for *A. fumigatus* isolates with a posaconazole MIC of 0.5 mg/L, can be considered [110]. 

Evidence concerning the optimal antifungal treatment of *Aspergillus* osteomyelitis, including FRI, will never be generated based on well-designed clinical trials. Therefore, sharing experiences via case reports is of absolute value, provided that standardized information on diagnosis, treatment details and clinical outcomes is documented. The development of an international registry of *Aspergillus* osteomyelitis cases might enable structured case documentation and enhance development of evidence-based diagnostic and treatment strategies. An analogous international registry has been established for rare invasive fungal diseases such as mucormycosis and hyalohyphomycosis (i.e., the FungiScope^TM^ registry) [111]. As infections caused by *Aspergillus* species are not included in the FungiScope^TM^ registry, there is a need for the development of a registry for *Aspergillus* bone infections, supported by international organizations.

The abovementioned methodological considerations of bone penetration studies hamper the interpretation of currently available plasma/bone concentration ratios. Furthermore, as bone exposure cannot be routinely monitored in clinical practice, it might be unattainable to address all methodological challenges. In our opinion, the relationship between plasma concentrations, as a surrogate for tissue exposure, and clinical outcomes should be further exploited. However, this approach is only valid if adequate bone penetration of the antifungal drug has been reliably demonstrated. Importantly, single bone concentration measurements are informative to confirm at least the penetration of the antifungal in bone.

## 6. Conclusions

We presented a patient with FRI caused by *A. fumigatus* who was treated with voriconazole and isavuconazole during six months. Although fracture union was not achieved during the following six months after therapy cessation, no confirmatory signs of FRI were observed. The efficacy of isavuconazole and other antifungal drugs in the treatment of *Aspergillus* FRI and other types of bone infection is unclear, as evidence is limited to case reports, which are characterized by methodological heterogeneity. Furthermore, data on bone penetration of antifungals are scarce and inconclusive. Standardized case documentation, based on consensus criteria for diagnosis and clinical outcome evaluation, is needed to establish evidence-based diagnostic and therapeutic strategies for *Aspergillus* bone infections, including FRI.

## Figures and Tables

**Table 1:** Demographic, clinical and therapeutic characteristics of *Aspergillus* fracture-related infections (*n* = 6).

Reference (Year)	Age, Sex	Infection Location	Infection Mechanism	Host Factors	*A.* Species	Surgical Treatment	AFT	Posology	Rationale Switch	Duration	TDM	Clinical Outcome	FU Clinical Outcome
Rodríguez-Hernández (2001) [3]	30,F	Parietal bone (R)	DI: fracture after cranial trauma	None	*A. fumigatus*	SD	AmB IV	1 mg/kg q24 h	NA	27 wk	Y	IC	2 yr after FRI diagnosis
							→ Itr PO	200 mg q12 h	NS				
Mouas (2005) [4]	46,M	Metacarpal bone (R)	DI: fracture after trauma	None	*A. terreus*	SD	Vor PO	150–200 mg q12 h	NA	93 d	N	IF	9 yr after trauma
Mouas (2005) [4]	43,M	Femur, fibula (L)	DI: fracture after trauma	None	*A. fumigatus*	SD, external fixation, knee arthrodesis	Itr	400 mg q24 h	NA	±35 wk	Y	IF	14 mo after trauma
							→ L-AmB IV	3 mg/kg q24 h	TF				
							→ Vor PO	200 mg q12 h (d1: 400 mg q12 h)	TF				
Garazzino (2008) [5]	69,M	Tibia (L)	DI: chronic infection after trauma (30 yr before)	DM	*A. flavus*	SD	Vor IV,PO	4 mg/kg q12 h (d1: 6 mg/kg q12 h)	NA	7 mo	N	IF	≥4 mo after AFT initiation
Dabkana (2015) [6]	26,F	Tibia, patella, femur (R)	DI: femur fracture after trauma	None	NS	Sequestrectomy, amputation, hip disarticulation	Tioconazole	NS	NA	NS	N	IC	NS
Takagi (2019) [7]	74,M	T11-T12 vertebrae	DI: fractures of T12, L1, rib 10–12 (L) after trauma	None	*A. terreus*	Partial laminectomy, spinal fusion	Vor IV,PO	600 mg/d	NA	5 mo	N	IC	2 yr after surgery

*A.*: *Aspergillus*; AFT: antifungal treatment; AmB: amphotericin B (formulation not specified); d: day; DI: direct inoculation; DM: diabetes mellitus; F: female; FRI: fracture-related infection; FU: follow-up; IC: inconclusive; IF: infection free; Itr: itraconazole; IV: intravenously; L: left; L-AmB: liposomal amphotericin B; M: male; mo: month; N: no; NA: not applicable; NS: not specified; PO: *per os*; R: right; SD: surgical debridement; TDM: therapeutic drug monitoring; TF: treatment failure; U: unknown; Vor: voriconazole; wk: week; Y: yes; yr: year; →: antifungal treatment switch.

**Table 2:** *Aspergillus* osteomyelitis cases with documented therapeutic drug monitoring for itraconazole, voriconazole or posaconazole (*n* = 18).

Reference (Year)	Antifungal Drug	Posology	Duration of AFT at Sampling Time	Time after Last Dose (Hours)	Plasma Concentration (mg/L)
Fracture-related infection					
Rodríguez-Hernández (2001) [3]	Itr PO	200 mg q12 h	NS	NS	2.26–2.29
Mouas (2005) ^a^ [4]	Itr	400 mg q24 h	NS	NS	Within applied therapeutic range (NS)
Sternal/rib osteomyelitis					
Vandecasteele (2002) ^b–f^ [23]	Itr PO	200 mg q12 h	NS	±12	Within therapeutic range (0.791–8.066)
Mouas (2005) ^g^ [4]	Itr	400 mg q24 h	NS	NS	Within applied therapeutic range (NS)
Asare (2013) [24]	Vor IV,PO	300 mg q12 h (d1: 500 mg q12 h)	3 d	NS	5
Assaf (2020) [25]	Vor IV,PO	4 mg q12 h (d1: 6 mg/kg q12 h)	≥7 d to ≤2 mo	NS	2.2–2.4
	Isa	200 mg q24 h (d1,2: 200 mg q8 h)	5 d	NS	3.2
Doub (2020) [26]	Pos PO	300 mg q24 h	2 wk	±24	0.9
		400 mg q24 h	6 wk ^h^	±24	2.1
Routray (2020) [27]	Vor IV,PO	200 mg q12 h	±4 wk	NS	Undetectable
	Vor PO	400 mg q12 h	±8 wk ^h^	NS	Undetectable
Osteomyelitis of the lower extremities					
Lodge (2004) [28]	Itr PO	200 mg q12 h	±15 d	NS	Undetectable
Denes (2007) [19]	Vor IV,PO	4 mg/kg q12 h (d1: 6 mg/kg q12 h)	1 d	±12	2.41
			2 d	±12	4.09
			NB: cortical and medullar bone concentrations on day 6: 1.9 μg/g and 20.3 μg/g		
Vertebral osteomyelitis					
Studemeister (2011) [15]	Vor IV	4 mg/kg q12 h	NS	±12	2.4
	Vor PO	200 mg q12 h	≥6 wk	±12	3.3
		150 mg q12 h	NS	±12	2.3
Spondylodiscitis					
Grandière-Perez (2000) [29]	Itr	800 mg/d	NS	NS	Mean concentration: 3
Takagi (2002) [30]	Itr PO	900 mg/d	19 d	NS	Itr + hydroxy-Itr: 5.9
		200 mg/d	26 d ^i^	NS	Itr + hydroxy-Itr: 13
Comacle (2015) [31]	Vor IV	4 mg q12 h (d1: 6 mg/kg q12 h)	10 d	NS	0.7 ^j^

AFT: antifungal treatment; d: day; Isa: isavuconazole; Itr: itraconazole; IV: intravenously; mo: month; NB: *nota bene*; NS: not specified; PO: *per os*; Pos: posaconazole; Vor: voriconazole; wk: week. ^a^ Case: 43-year old male patient (as described in Appendix C). ^b–f^ Cases: 74-year old female patient, 76-year old male patient, 69-year old female patient, 75-year old female patient (no host factors), 75-year old female patient (immunosuppressive therapy) (as described in Appendix C). ^g^ Case: 31-year old male patient (as described in Appendix C). ^h^ Four weeks after dosage increase to 400 mg q12 h. ^i^ Five days after dosage reduction to 200 mg/day. ^j^ Regular therapeutic drug monitoring was performed during oral voriconazole therapy, but no specifications were given on sampling time and measured plasma concentrations.

**Figure A1:**
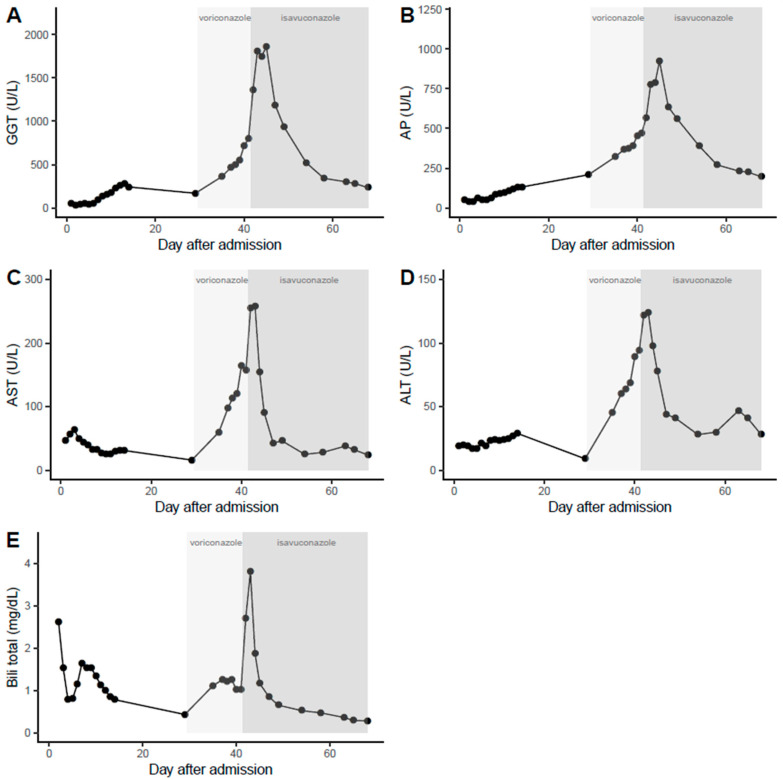
(**A**) Gamma-glutamyl transferase (GGT) levels in function of the time after hospital admission; (**B**) Alkaline phosphatase (AP) levels in function of the time after hospital admission; (**C**) Aspartate aminotransferase (AST) levels in function of the time after hospital admission; (**D**) Alanine aminotransferase (ALT) levels in function of the time after hospital admission; (**E**) Total bilirubin (bili total) levels in function of the time after hospital admission.

## Data Availability

All data relevant to the case report and literature review are included in the manuscript. Additional data are available from the authors upon reasonable request.

## Appendix A

Graphical presentation of liver function parameters of the 77-year-old male patient with *Aspergillus fumigatus* FRI, described in our case report.

## Appendix B

Terminology for the variables described in the literature review on antifungal treatment of *Aspergillus* osteomyelitis (Table 1 and Appendix C).

The infection type was classified into five categories: (1) FRI, (2) sternal/rib osteomyelitis, (3) osteomyelitis of the lower extremities (not related to a fracture or trauma), (4) vertebral osteomyelitis and (5) spondylodiscitis. The following classification was used for the infection mechanism: (1) contiguous spread from an adjacent focus of invasive aspergillosis or haematogenous spread from a distant focus of invasive aspergillosis, (2) direct inoculation of conidia (e.g., traumatic or iatrogenic), (3) not specified (i.e., infection mechanism was not specified by the authors) (4) unknown (i.e., infection mechanism was unknown according to the authors).

Included host factors were (1) diabetes mellitus, (2) graft-versus-host disease (GVHD), (3) human immunodeficiency virus infection, (4) haematologic malignancy, (5) inherited severe immunodeficiency, (6) immunosuppressive therapy (i.e., T- and B-cell immunosuppressants, prolonged use of systemic corticosteroids), (7) neutropenia, (8) allogeneic stem cell transplantation and (9) solid organ transplantation. GVHD, haematologic malignancy, immunodeficiency, immunosuppressive therapy, neutropenia, stem cell transplantation and solid organ transplantation were defined according to the documented host factors for probable invasive fungal diseases by the European Organization for Research and Treatment of Cancer and the Mycoses Study Group Education and Research Consortium (EORCT/MSGERC) [1]. Neutropenia related to haematologic malignancy, allogeneic stem cell transplantation, solid organ transplantation or immunosuppressive therapy and immunosuppressive therapy related to haematologic malignancy, allogeneic stem cell transplantation, solid organ transplantation or GVHD were not classified separately.

The rationale for antifungal treatment switch or discontinuation was documented if applicable and classified as follows: (1) poor adherence to treatment, (2) resistance/reduced susceptibility of the isolated *Aspergillus* species, (3) sub- or supratherapeutic drug plasma concentrations, (4) toxicity/adverse drug reactions, (5) treatment failure or disease progression despite antifungal therapy (and surgical treatment).

The clinical outcome was documented according to the following criteria: (1) death (probably) related to invasive aspergillosis, (2) death (probably) not related to invasive aspergillosis, (3) infection free (i.e., no (recurrence of) infection as explicitly described by the authors or—in case of FRI—absence of confirmatory infection criteria according to the consensus definition of FRI [2]), (4) not infection free (i.e., signs of (recurrent) infection as explicitly described by the authors or—in case of FRI—presence of confirmatory criteria), (5) inconclusive (i.e., infection status is not clear based on the information given in the case report), (6) not specified (i.e., clinical outcome is not specified by the authors).

## Appendix C

**Table A1:** Demographic, clinical and therapeutic characteristics of *Aspergillus* osteomyelitis and spondylodiscitis cases (*n* = 108).

Reference (Year)	Age, Sex	Infection Location	Infection Mechanism	Host Factors	*A.* Species	Surgical Treatment	AFT	Posology	Rationale Switch	Duration	TDM	Outcome	FU Clinical Outcome
Fracture-related infection													
Rodríguez-Hernández (2001) [3]	30,F	Parietal bone (R)	DI: fracture after cranial trauma	None	*A. fumigatus*	SD	AmB IV	1 mg/kg q24 h	NA	27 wk	Y	IC	2 yr after FRI diagnosis
							→ Itr PO	200 mg q12 h	NS				
Mouas (2005) [4]	46,M	Metacarpal bone (R)	DI: fracture after trauma	None	*A. terreus*	SD	Vor PO	150–200 mg q12 h	NA	93 d	N	IF	9 yr after trauma
	43,M ^a^	Femur, fibula (L)	DI: fracture after trauma	None	*A. fumigatus*	SD, external fixation, knee arthrodesis	Itr	400 mg q24 h	NA	±35 wk	Y	IF	14 mo after trauma
							→ L-AmB IV	3 mg/kg q24 h	TF				
							→ Vor PO	200 mg q12 h (d1: LD)	TF				
Garazzino (2008) [5]	69,M	Tibia (L)	DI: chronic infection after trauma (30 yr before)	DM	*A. flavus*	SD	Vor IV,PO	4 mg/kg q12 h (d1: LD)	NA	7 mo	N	IF	≥4 mo after AFT initiation
Dabkana (2015) [6]	26,F	Tibia, patella, femur (R)	DI: femur fracture after trauma	None	NS	Sequestrectomy, amputation, hip disarticulation	Tioconazole	NS	NA	NS	N	IC	NS
Takagi (2019) [7]	74,M	T11-T12 vertebrae	DI: fractures of T12, L1 vertebrae, rib 10–12 (L) after trauma	None	*A. terreus*	Partial laminectomy, spinal fusion	Vor IV,PO	600 mg/d	NA	5 mo	N	IC	2 yr after surgery
Sternal/rib osteomyelitis													
Allen (2002) [44]	67,F	Sternum	C/H: IPA (8 yr before)	None	*A. fumigatus*	None	L-AmB IV	1 mg/kg q24 h	NA	10 wk	N	D **	8 mo after AFT cessation
							→ Itr PO	200 mg q12 h	TA				
Vandecasteele (2002) [23]	74,F ^b^	Sternum	DI: CABG	None	*A. flavus*	SD	Itr PO	200 mg q12 h	NA	128 d	Y	IC	≥128 d after AFT initiation
	76,M ^c^	Sternum	DI: CABG	None	*A. flavus*	Curettage	Itr PO	200 mg q12 h	NA	87 d	Y	IC	≥87 d after AFT initiation
	69,F ^d^	Sternum	DI: CABG	IS	*A. flavus*	Multiple SDs, marsupiali-zation of abscess	Itr PO	200 mg q12 h	NA	100 d	Y	IC	≥100 d after AFT initiation
	75,F ^e^	Sternum	DI: CABG	None	*A. flavus*	Multiple curettages	Itr PO	200 mg q12 h	NA	122 d	Y	IC	≥122 d after AFT initiation
	75,F ^f^	Sternum	DI: CABG	IS	*A. flavus*	Curettage	Itr PO	200 mg q12 h	NA	11 d	Y	D *	55 d after OM diagnosis
Elahi (2005) [45]	62,M	Sternum, rib 6–10 (R)	DI: repeated CABG	DM	*A. fumigatus*	Multiple SDs, partial resection of sternum & ribs 6–8	Itr PO	200 mg/d	NA	6 mo	N	IC	12 mo after surgery
Mouas (2005) [4]	31,M^g^	Rib (R) (NS)	C/H: IPA	HIV	*A. fumigatus*	None	AmB IV	1 mg/kg q24–96 h	NA	±17 mo	Y	D **	±21 mo after OM diagnosis
							→ AmB IV	1 mg/kg q72–96 h	NS				
							+5-FC	NS					
							→ Itr	400 mg q24 h	TF				
							→ AmB IV	NS	TF				
							→ Vor PO	100–200 mg q12 h	TF				
	55,M	Sternum	NS	SOT	*A. fumigatus*	NS	AmB IV	NS	NA	≥17 d	N	IC	≥17 d after Vor initiation
							+Itr	NS					
							→ Vor PO	NS	NS				
Soto-Hurtado (2005) [46]	65,M	Lower ribs (L) (NS)	C/H: pulmonary aspergillomas	None	U	Thoracotomy	AmB IV	NS	NA	NS	N	D *	Few days after AFT initiation
Natesan (2007) [47]	29,F	Sternum	DI: pericardiectomy	SOT, DM	*A. terreus*	SD	Itr PO	200 mg q24 h	NA	±2–4 mo	N	IF	±6 mo after pericard-iectomy
							→ L-AmB IV	150 mg q24 h	NS				
							→ Caf IV	50 mg q24 h (d1: LD)	R				
							→ Vor	200 mg q24 h	NS				
Verghese (2008) [48]	70,M	Rib 7 (L)	DI: CABG	DM	*A. flavus*	Partial rib excision, abscess drainage	Vor	200–400 mg q12 h	NA	5 mo	N	IC	NS
Horn (2009) [49]	62,F	Rib (NS)	NS	None	*A. fumigatus*	Surgery type NS	Vor	NS	NA	16 d	N	IF	71 d after OM diagnosis
	63,M	Sternum	DI: aortic valve replacement	None	*A. fumigatus*	Surgery type NS	Flu	NS	NA	10 d	N	D	33 d after OM diagnosis
	48,F	Sternum	DI: aortic valve replacement	None	*A. fumigatus*	Surgery type NS	Vor	NS	NA	26 d	N	IF	26 d after OM diagnosis
Puri (2011) [50]	52,M	Rib (NS) (R)	C/H: suspicion of IPA	None	U	Partial resection of sternum, 4 ribs & lung lobe, chest wall reconstruction	AmB IV	NS	NA	NS	N	NS	NS
Asare (2013) [24]	69,M	Sternum	DI: CABG, aortic valve replacement	None	*A. fumigatus*	Sternectomy, multiple SDs	Vor IV,PO	300 mg q12 h (d1: LD)	NA	NS	Y	IC	NS
Landaburu (2019) [51]	61,F	Rib 10,12 (R)	DI: pulmonary segmentectomy	IS	*A. flavus*	None	Vor IV,PO	200 mg q12 h	NA	8 mo	N	IC	8 mo after AFT initiation
Assaf (2020) [25]	65,M	Sternum	DI: heart Tx, re-opening of sternotomy	SOT	*A. fumigatus*	Wire removal, abscess drainage	Vor IV,PO	4 mg q12 h (d1: LD)	NA	12 mo	Y	IF	≥10 mo after Isa initiation
							→ L-AmB IV	3 mg/kg q24 h	TA				
							→ Isa	200 mg q24 h (d1,2: LD)	TA				
Doub (2020) [26]	37,M	Rib 10 (R)	U	None	*A. fumigatus*	Multiple SDs, removal of rib graft, decortication, chest wall reconstruction	Vor IV,PO	4 mg/kg (d1: LD)	NA	9 mo	Y	IF	9 mo after AFT cessation
							Pos PO	300–400 mg q24 h	TA				
Routray (2020) [27]	65,F	Sternum	DI: CABG	DM	*A. fumigatus*	Partial sternectomy, abscess drainage, multiple SDs	Mcf IV	100 mg q24 h	NA	±7 mo	Y	IC	≥2 wk after AFT reinitiation
							+Vor IV,PO	200 mg q12 h					
							→ Vor PO	200–400 mg q12 h	NS				
							→ Isa PO	372 mg q24 h (d1,2: LD)	AD, TA				
							→ L-AmB IV	400 mg q24 h	AD, TA	±9.5 mo (re-initiation 6 mo after Isa discont-inuation)			
							+Vor IV	400 mg q12 h					
							+Mcf IV	100 mg q24 h					
							→ Vor IV,PO	200–400 mg q12 h	TA				
							+Mcf IV	100 mg q24 h					
							→ Vor PO	200 mg q12 h	NS				
Osteomyelitis of the extremities													
Kaneko (2002) [52]	57,F	Femur (R)	U	SOT	*A. fumigatus*	None	AmB IV	0.7 mg/kg q24 h	NA	13 mo	N	IC	±1 yr after OM diagnosis
							+5-FC	120 mg/kg q24 h					
							→ Itr	200 mg/d	NS				
Lodge (2004) [28]	64,M	Calcaneal bone (R)	C/H: IPA	SOT	*A. fumigatus*	SD, partial calcanectomy	Itr PO	200 mg q12 h	NA	±13 mo	Y	IF	12 mo after AFT cessation
							+ABLC IV	5 mg/kg q24 h					
							+AmB INH	25 mg/wk					
							→ ABLC IV	5 mg/kg q24 h	ST				
							+AmB INH	25 mg/wk					
							→ Pos PO	400 mg q12 h	TA, TF				
							NB: prior AFT (AmB INH ± Itr) for IPA						
Brodsky (2005) [53]	40,M	5th distal phalanx (R)	DI: paronychia after toenail clipping	HM	*A. versicolor*	SD, partial resection of phalanx	ABLC IV	NS	NA	±6 wk	N	D **	2 mo after AFT cessation
							→ Itr PO	NS	NS				
							NB: prior empiric AFT (Flu + AmB IV)						
Denes (2007) [19]	83,F	Femur, tibia (L)	DI: knee arthritis after CS infiltration	None	*A. fumigatus*	SD, amputation	Vor IV,PO	4 mg/kg q12 h (d1: LD)	NA	<1 wk	Y	D *	Few days after amputation
Hodiamont (2009) [54]	18,M	Cuneiform bones (R)	NS	ID	*A. fumigatus*	SD	Vor IV	4 mg/kg q12 h	NA	±13.5 mo	N	IF	±13.5 mo after AFT initiation
							→ Caf IV	70 mg q24 h	RS				
							→ Pos PO	400 mg q12 h	NS				
							NB: Itr prophylaxis started at age of 7 yr and restarted after Pos cessation						
Horn (2009) [49]	60,F	Scapula (R)	C/H: IPA	SOT	*A. fumigatus*	None	Vor	NS	NA	69 d	N	D	74 d after OM diagnosis
							→ Pos	NS	NS				
							+Mcf IV	NS					
Karia (2011) [55]	79,F	Femur	U	HM	*A. fumigatus*	None	Vor IV	NS	NA	4 wk	N	IC	4 wk after AFT initiation
							+AmB IV	NS					
Hall (2012) [56]	72,F	Proximal humerus (R)	DI: prior shoulder arthroscopy	DM	*A. fumigatus*	SD, reverse shoulder arthroplasty	Vor PO	200 mg q12 h	NA	16 mo	N	IF	2 yr after OM diagnosis
							+AmB cement spacer	300 mg		±6–12 wk			
Hébert-Seropian (2020) [57]	52,M	Scapula (L)	C/H: IPA	SOT, GVHD	*A. fumigatus*	Multiple SDs & drainages	Vor IV,PO	NS	NA	±22 mo	N	IC	2 yr after 2nd surgery
Vertebral osteomyelitis													
van Ooij (2000) [58]	56,F	T12-L1	NS	HM	*A. fumigatus*	SD, laminectomy, spinal stabilization	AmB IV	Total dose: 3000 mg	NA	≥2.5 mo	N	IC	≥5 yr after OM diagnosis
							→ Itr PO	NS	NS				
Frazier (2001) [59]	52,M	T7-T8,L1-L3	C/H: *A.* endocarditis	IS	*A. fumigatus*	None	AmB IV	Total dose: 2000 mg	NA	NS	N	D *	33 d after surgery
	62,M	L2-L3	U	IS	*A. fumigatus*	Corpectomy, spinal stabilization	AmB IV	Total dose: 1100 mg	NA	NS	N	IF	≥18 mo after OM diagnosis
Govender (2001) [60]	54,M	L4	U	None	*A. fumigatus*	Decompression, spinal stabilization	AmB IV	0.25–0.7 mg/kg q24 h	NA	6–9 wk	N	IF	≥2 yr after OM diagnosis
							+5-FC PO	200–400 mg/kg q24 h					
	29,F	T1	U	None	*A. fumigatus*	Decompression, spinal stabilization	AmB IV	0.25–0.7 mg/kg q24 h	NA	6–9 wk	N	IF	≥2 yr after OM diagnosis
							+5-FC PO	200–400 mg/kg q24 h					
	18,F	T7	U	None	*A. fumigatus*	Decompression, spinal stabilization	AmB IV	0.25–0.7 mg/kg q24 h	NA	6–9 wk	N	IF	≥2 yr after OM diagnosis
							+5-FC PO	200–400 mg/kg q24 h					
	33,M	T1	U	None	*A. fumigatus*	Decompression, spinal stabilization	AmB IV	NS	NA	6–9 wk	N	IF	≥2 yr after OM diagnosis
							+5-FC PO						
							→ Flu	400 mg/d	TA				
Chi (2003) [61]	63,M	C2-C5	U	DM	*A. flavus*	Laminectomy	Itr PO	200 mg q8–12 h	NA	2 wk	N	D *	2 wk after surgery
							→ AmB IV	25 mg q24 h	NS				
Salvalaggio (2003) [62]	46,M	L3	U	SOT, DM	*A. fumigatus*	SD, discectomy	AmB IV	1 mg/kg q24 h	NA	16 wk	N	IF	≥18 mo after Tx
							→ AmB IV	1 mg/kg q24 h	TF				
							+5-FC	6 g q24 h					
							→ AmB IV	1 mg/kg q24 h	NS				
							→ Caf IV	50 mg q24 h	TA				
Stratov (2003) [63]	52,M	L2,L4	U	None	*A. fumigatus*	Partial vertebrectomy, discectomy	D-AmB IV	1 mg/kg q24 h	NA	±6.5 mo	N	IC	≥15 mo after AFT cessation
							→ L-AmB IV	4–7.5 mg/kg q24 h	TA, TF				
							→ Vor IV,PO	200–280 mg q12 h	TF				
Vaishya (2003) [64]	35,F	D11	U	None	NS	Corpectomy, spinal stabilization	AmB IV	NS	NA	±6 wk	N	D **	2 mo after surgery
							→ AmB IV	NS	NS				
							+Itr PO	200 mg q12 h					
Kim (2004) [112]	68,M	T1-T3	C/H: suspicion of IPA	None	NS	Laminectomy, abscess drainage	Itr	400 mg/d	NA	27 d	N	D *	≥5 wk after surgery
Salloum (2004) [65]	48,M	T6-T7, rib 9–10 (L)	H: IV drug use	None	*A. fumigatus*	Rib resection, abscess drainage, chest wall excision	Itr PO	200 mg q12 h	NA	18 mo	N	IF	18 mo after AFT initiation
Santos (2004) [66]	59,F	T11-T12	U	HM	*A. fumigatus*	Arthrodesis, partial vertebrectomy	Itr	200 mg q12 h	NA	9 mo	N	IC	NS
							→ Itr	200 mg q12 h	NS				
							+L-AmB IV	3 mg/kg q24 h					
							→ Itr	200 mg q12 h	NS				
Nusair (2005) [67]	49,F	T8-T9	NS	None	*A. fumigatus*	SD, corpectomy, discectomy, arthrodesis	Vor IV,PO	200 mg q12 h	NA	6 mo	N	IF	±2 mo after surgery
Myhre (2006) [68]	57,M	L2-L5	DI: posterior spinal fusion	None	*A. fumigatus*	SD	Vor	NS	NA	NS	N	IC	NS
Dayan (2007) [69]	78,F	T12-L1	NS	IS	*A. fumigatus*	Spinal stabilization & realignment	Vor	NS	NA	NS	N	D **	2 wk after surgery
Andaluz (2008) [70]	65,M	T1-T6	C/H: IPA 3 yr before	None	*A. fumigatus*	None	Vor PO	400 mg/d	NA	≥5.5 mo	N	IC	≥4 mo after OM diagnosis
							→ Caf IV	50 mg q24 h (d1: LD)	NS				
							→ D-AmB IV	1 mg/kg q24 h	TF				
							→ L-AmB IV	NS	TA				
							→ Pos PO	800 mg/d	TF				
Horn (2009) [49]	58,M	T7-T8	NS	SOT	*A. fumigatus*	None	Vor	NS	NA	≥84 d	N	NIF	84 d after OM diagnosis
							+Caf IV	NS					
							→ Vor	NS	NS				
	44,F	T8-T9	NS	None	*A. fumigatus*	Surgery type NS	Vor	NS	NA	≥84 d	N	NIF	84 d after OM diagnosis
	46,F	T8-T9	U	None	*A. flavus*	Surgery type NS	Vor	NS	NA	≥84 d	N	NIF	90 d after OM diagnosis
	48,F	T4	NS	None	*A. fumigatus*	Surgery type NS	L-AmB IV	NS	NA	≥84 d	N	NIF	84 d after OM diagnosis
							→ Vor	NS	NS				
Tew (2009) [71]	50,M	T2-T8	U	DM	*A. fumigatus*	Laminectomy, costovertebral joint excision, abscess drainage	Vor IV	4 mg/kg q12 h (d1: LD)	NA	±2 wk	N	D *	2 wk after surgery
							Vor PO	200 mg q12 h					
Nandeesh (2010) [72]	66,F	L2-S1	U	DM	U	SD, anterior decompression, spinal stabilization	Vor	NS	NA	NS	N	IC	NS
Batra (2011) [73]	45,M	L3-L5	U	None	*A. fumigatus*	SD, laminectomy	Itr PO	200 mg q12 h	NA	3 mo	N	IC	FU: 36 mo
Studemeister (2011) [15]	52,F	L2-L3,L4-L5	C/H: pulmonary aspergillomas	None	*A. fumigatus*	SD, laminectomy, discectomy, internal fixation	Vor IV	4 mg/kg q12 h	NA	6 mo	Y	IF	2 mo after initial surgery
							Vor PO	150–200 mg q12 h					
Zhu (2011) [74]	46,M	L4-L5	C/H: IPA	SOT	*A. flavus*	SD, spinal stabilization	Vor IV,PO	NS	NA	15 mo	N	IC	12 mo after AFT cessation
							→ ABCD IV	NS	NS				
							→ Itr IV	200 mg q12–24 h	TA				
							→ Itr IV,PO alternately	200 mg q12–24 h	NS				
							→ Itr PO	200 mg q24 h	NS				
							NB: prior AFT (Mcf + ABCD + Vor) for IPA						
Sethi (2012) [75]	25,M	L4-L5	U	None	U	Surgical decompression, interbody fusion	Itr	200 mg q12 h	NA	3 mo	N	IC	FU: 1 yr
	19,M	D10-D11	U	None	U	Corpectomy, spinal fusion	Itr	200 mg q12 h	NA	2 mo (ITT)	N	IC	No FU
Jiang (2013) [76]	40,F	T1-T3	U	None	*A. nidulans*	SD, laminectomy	Vor IV	4 mg/kg q12 h (d1: LD)	NA	±1,5 mo	N	IC	1 yr after AFT cessation
							Vor IV	4 mg/kg q12 h (d1: LD)	AD	±6 mo (re-initiation after cessation)			
							→ Vor PO	200 mg q12 h	NS				
Nicolle (2013) [77]	65,M	C2-C3	C/H: prior otogenic skull base OM	DM	*A. flavus*	None (prior mastoidectomy)	Vor	NS	NA	12 mo	N	IC	12 mo after AFT initiation
McCaslin (2015) [78]	19,F	T12-L1	NS	HM	NS	Laminectomy, abscess drainage	Vor IV	4 mg/kg q12 h (d1: LD)	NA	NS	N	D *	NS
Yoon (2015) [79]	53,M	L2-L3	U	None	NS	Laminectomy, corpectomy, spinal fusion	AmB IV	0.35 mg/kg q24 h	NA	30 d	N	IC	7 mo after discharge
Li (2016) [80]	53,M	L2-L3,L5	C/H: suspicion of IPA	None	U	Multiple SDs & drainages, decompression, spinal stabilization	Vor IV,PO	4 mg/kg q12 h (d1: LD)	NA	≥3 mo	N	IC	≥3 mo after AFT initiation
Ono (2018) [81]	70,F	T4-T5	C/H: IPA	HM	*A. fumigatus*	None	L-AmB IV	2.5 mg/kg q24 h	NA	14 d	N	D *	14 d after admission
							→ L-AmB IV	4 mg/kg q24 h	NS				
							+Mcf IV	200 mg q24 h					
							→ Vor IV	4 mg/kg q12 h (d1: LD)	NS				
Shweikeh (2018) [82]	58,F	L4-S1	DI: multiple spinal surgeries, epidural injections	IS	NS	None	Vor IV	NS	NA	3 mo (ITT)	N	IC	NS
							+Mcf IV	NS					
Yang (2019) [83]	51,M	T5-T10	C/H: fungal infection (NS)	None	*A. fumigatus*	Laminectomy	AmB IV	60 mg q24 h	NA	NS	N	D *	NS
Karaisz (2020) [84]	64,F	Lumbar spine	NS	SOT	*A. fumigatus*	None	AmB IV	NS	NA	±2 mo	N	D *	±2 mo after AFT initiation
							→ Isa	NS	NS				
							+anidulafungin	NS					
Senosain-Leon (2020) [85]	29,M	T4-T6	C/H: IPA	HIV	NS	None	D-AmB	NS	NA	2 d	N	D *	2 d after D-AmB initiation
							NB: prior AFT (Itr PO) for IPA						
Spondylodiscitis													
Grandière-Perez (2000) [29]	40,M	L3-L4	C/H: IPA	HM	*A. terreus*	NS	None	NA	NA	NA	Y	D **	6 mo after spondylo-discitis diagnosis
							NB: prior treatment for IPA: AmB IV → Itr	2 mg/kg q24 h → 800 mg/d	NA	≥1 mo			
Park (2000) [86]	37,M	L3- -S1	U	HM	*A. terreus*	SD, spinal fusion	AmB IV	Total dose: 2000 mg	NA	NS	N	IF	NS
Tang (2000) [87]	43,M	L2-L3, L4-L5	U	SOT	*A. flavus*	None (NB: Girdlestone procedure for *A.* coxarthritis)	L-AmB IV	5 mg/kg q24 h	NA	≥15 wk	N	IC	1 yr after Tx
							→ Itr	400 mg/d	NS				
van Ooij (2000) [58]	45,M	T4-T5	C/H: pulmonary aspergillomas	HM	NS	SD, spinal stabilization	AmB IV	Total dose: 3800 mg	NA	±6.5 mo	N	IC	3 yr after surgery
							→ Itr PO	NS	NS				
							NB: prior AFT (AmB) for pulmonary aspergillomas						
	69,M	T12-L1	C/H: suspicion of IPA	HM	*A. fumigatus*	SD, spinal stabilization	AmB IV	Total dose: 2070 mg	NA	≥6 wk	N	D **	4 mo after surgery
							+5-FC IV	NS					
							→ Itr	400 mg/d	NS				
							NB: prior AFT (AmB + Itr) for suspicion of IPA						
	39,F	L4-L5	C/H: IPA	HM	*A. fumigatus*	SD, spinal stabilization	AmB IV	NS	NA	≥4 mo	N	D **	4 mo after surgery
							→ Itr PO	400 mg/d	NS				
							→ Experi-mental AFT	NS	TF				
							→ AmB IV	NS	TF				
Beckers (2002) [88]	72,F	T11-T12	C/H: IPA	None	NS	Vertebrectomy	AmB IV	NS	NA	NS	N	D **	NS
							→ Itr	NS	TA				
							NB: prior AFT (Itr) for IPA						
Takagi (2002) [30]	51,M	L1-L2	U	HM	NS	None	AmB IV	50 mg q24 h	NA	≥40 d	Y	IC	NS
							+Itr	200 mg/d					
							→ Itr PO	200–900 mg/d	TA				
							NB: prior AFT (AmB + Itr) for suspected fungal infection						
Lenzi (2004) [89]	50,F	L4-L5	DI: spinal surgery	None	*A. fumigatus*	SD	Itr	NS	NA	NS	N	IC	3 mo after surgery
Park (2004) [90]	59,F	L2-L4	U	SOT	NS	Surgical decompression	AmB IV	NS	NA	NS	N	D *	≥60 d after diagnosis
Mouas (2005) [4]	76,M	C5-T2	U	None	*A. fumigatus*	None	D-AmB IV	Total dose: 1200 mg	NA	≥14 mo	N	IF	±2.5–3 yr after diagnosis
							→ Vor IV,PO	≥150 mg q12 h	NS				
							NB: prior empiric AFT (Itr)						
	62,M	NS	NS	None	*A. fumigatus*	NS	AmB IV	NS	NA	≥84 d	N	NIF	≥84 d after Vor initiation
							+5-FC	NS					
							→ Vor IV,PO	NS	NS				
	53,F	Lumbar spine	NS	SOT	*A. versicolor*	NS	AmB IV	NS	NA	≥197 d	N	NIF	≥197 d after Vor initiation
							→ Itr	NS	NS				
							→ Vor PO	NS	NS				
	45,M	L3-L4	U	None	*A. terreus*	NS	Vor PO	NS	NS	127 d	N	IC	≥127 d after Vor initiation
	39,M	L4-L5	U	HM	*A. fumigatus*	NS	AmB IV	NS	NA	≥47 d	N	IC	≥47 d after Vor initiation
							→ Itr	NS	NS				
							→ Vor PO	NS	NS				
	69,M	Lumbar spine	NS	DM	*A. fumigatus*	NS	AmB IV	NS	NA	≥6 d	N	IC	≥6 d after Vor initiation
							→ Vor IV	NS	NS				
Kolbe (2007) [91]	51,F	L4-L5 (+menin-geal involve-ment)	DI: discography, epidural steroid injections	None	*A. fumigatus*	None	Caf IV	NS	NA	NS	N	D *	4–5 mo after spondylo-discitis diagnosis
							+Vor	NS					
							→ Long-term AF (NS)	NS	NS				
Wéclawiak (2007) [92]	18,M	T12-L1	U	SOT	*A. fumigatus*	None	Vor	200–400 mg/d	NA	4 mo	N	IC	4 mo after AFT initiation
Gerlach (2009) [93]	25,M	L2-L3	C/H: IPA	SOT	*A. fumigatus*	SD, spinal stabilization	Vor	NS	NA	≥2 mo	N	IC	15 mo after surgery
							→ Vor	NS	TF				
							+Caf IV	50 mg q24 h					
							+L-AmB IV	5 mg/kg q24 h					
Oh (2009) [94]	46,M	L3-L5, C4-C5, D2-D4, D6-D7, D10-D11	C/H: pulmonary aspergillomas	SOT	NS	Decompression, interbody fusion	AmB IV	50 mg q24 h	NA	±15.5 mo	N	IC	±3 yr after admission
							→ Itr	100 mg q12 h	TF				
							→ Vor	200 mg q12 h	TF				
Ersoy (2011) [95]	46,M	T8-T9, L2-L3	C/H: IPA	SOT	*A. fumigatus*	Corpus resection, discectomy, abscess drainage	Vor	200 mg/d	NA	107 d	N	IC	≥18 mo after diagnosis
							NB: prior AFT (Caf) for IPA						
Li (2012) [96]	44,M	L4-L5, L5-S1	U	SOT, DM	*A. flavus*	SD, decompression, spinal fusion	Vor IV	4 mg/kg q12 h (d1: LD)	NA	NS	N	IC	≥20 wk after AFT initiation
							→ Vor PO	200 mg q12 h					
Raj (2013) [97]	45,F	L5-S1	NS	DM	*A. fumigatus*	Laminectomy, abscess drainage	Itr PO	200 mg q12 h	NA	3 mo	N	IC	9 mo after surgery
Shashidhar (2014) [98]	33,F	L2-L3	DI: spinal anesthesia	None	*A. fumigatus*	SD, discectomy, spinal fusion	Vor IV,PO	200 mg q12 h	NA	12 wk	N	IC	FU: 1 yr
Comacle (2015) [31]	20,M	T7-T12	DI: motorbike accident (3 yr before)	None	*A. niger*	SD, spinal arthrodesis	Vor IV	4 mg/kg q12 h (d1: LD)	NA	≥2 mo	Y	IC	4 mo after AFT initiation
							→ Vor PO	200–400 mg q12 h					
							→ Caf IV	50 mg q24 h (d1: LD)	TA				
							→ Vor PO	NS	NS				
							+Caf IV	50 mg q24 h					
							→ Vor PO	NS	NS				
Dai (2020) [99]	67,M	T3-T5	C/H: IPA	None	*A. fumigatus*	None	Vor	200 mg q12 h	NA	20 wk	N	IC	FU: 20 mo
	68,M	T12-L2	NS	IS	*A. fumigatus*	SD, laminectomy, decompression, instrumentation	Vor	200 mg q12 h	NA	16 wk	N	IC	FU: 24 mo
	50,F	L3-L4	NS	None	*A. fumigatus*	SD, laminectomy, instrumentation	Vor	200 mg q12 h	NA	18 wk	N	IC	FU: 24 mo
	48,M	L4-L	U/DI: minimally invasive spinal surgery	None	*A. fumigatus*	SD, laminectomy, instrumentation	Vor	200 mg q12 h	NA	16 wk	N	IC	FU: 15 mo
	43,M	L4-L5	U/DI: spinal surgery	None	*A. niger*	SD, laminectomy, decompression, instrumentation	Vor	200 mg q12 h	NA	22 wk	N	IC	FU: 20 mo
	66,M	L2-L3	U/DI: spinal surgery	None	NS	SD, laminectomy, instrumentation	Vor	200 mg q12 h	NA	20 wk	N	IC	FU: 18 mo
Fan (2020) [100]	49,M	T12-L1, L2-L3	NS	None	*A. flavus*	Percutaneous transforaminal endoscopic discectomy	Itr PO	150 mg q12 h	NA	13 mo	N	IC	9 mo after surgery
							+intervertebral Itr injections	NS					
Perna (2021) [12]	76,M	L2-L3	U	HM	*A. fumigatus*	None	L-AmB IV	NS	NA	7 mo	N	IF	7 mo after AFT initiation
							→ Vor PO	NS	NS				

5-FC: 5-fluorocytosine; *A.*: *Aspergillus*; →: antifungal treatment switch; ABCD: amphotericin B colloidal dispersion; ABLC: amphotericin B lipid complex; AD: poor treatment adherence; AFT: antifungal treatment; AmB: amphotericin B (formulation not specified); CABG: coronary artery bypass graft; Caf: caspofungin; C/H: contiguous/haematogenous; CS: corticosteroids; d: day; D: death; D-AmB: amphotericin B deoxycholate; DI: direct inoculation; DM: diabetes mellitus; F: female; Flu: fluconazole; FRI: fracture-related infection; FU: follow-up; GVHD: graft-versus-host disease; HIV: human immunodeficiency virus infection; HM: haematologic malignancy; IC: inconclusive; ID: immunodeficiency; IF: infection free; INH: inhalation; IPA: invasive pulmonary aspergillosis; IS: immunosuppressive therapy; Isa: isavuconazole; Itr: itraconazole; ITT: intention to treat; IV: intravenously; L: left; L-AmB: liposomal amphotericin B; LD: loading dose; M: male; Mcf: micafungin; mo: month; N: no; NA: not applicable; NB: nota bene; NIF: not infection free; NS: not specified; OM: osteomyelitis; PO: per os; Pos: posaconazole; R: right; RS: resistance/reduced susceptibility; SD: surgical debridement; SOT: solid organ transplantation; ST: sub- or supratherapeutic plasma concentrations; TA: toxicity/adverse drug reactions; TDM: therapeutic drug monitoring; TF: treatment failure; Tx: transplantation; U: unknown; Vor: voriconazole; wk: week; Y: yes; yr: year D *: death probably related to invasive aspergillosis; D **: death probably not related to invasive aspergillosis; D: cause of death not specified Loading doses for Caf IV: 70 mg q24 h on day 1, for Isa IV/PO: 200 mg q8 h on day 1 and 2 and for Vor IV/PO: 6 mg/kg q12 h on day 1, unless specified otherwise.^a–g^ Cases with documented therapeutic drug monitoring for itraconazole, voriconazole or posaconazole, as described in Table 2.
